# Wernicke’s Encephalopathy

**DOI:** 10.7759/cureus.3187

**Published:** 2018-08-22

**Authors:** Smit Patel, Karan Topiwala, Lawrence Hudson

**Affiliations:** 1 Neurology, Hartford Hospital, Hartford, USA

**Keywords:** wernicke’s encephalopathy, thiamine, alcohol toxicity, delirium, sun downing, vitamin b1, 5 fu infusion, stroke prevention

## Abstract

Wernicke’s encephalopathy (WE) is a neurologic emergency that requires immediate attention to prevent permanent neurological morbidity and mortality. It presents with confusion, ophthalmoplegia and gait ataxia which together comprise its classic triad. Thiamine deficiency related to alcohol abuse remains the primary culprit; non-alcoholic WE, however, can have an atypical clinical presentation and is often missed. Thus, although the diagnosis of WE remains primarily clinical, neuroimaging plays an important role, especially in the diagnosis of non-alcoholic WE. Here, we present a case of non-alcoholic WE with an atypical clinical presentation but typical magnetic resonance imaging (MRI) findings in a woman with a history of non-bariatric gastrointestinal surgery. Thiamine replacement therapy rapidly reversed her neurologic symptoms and MRI findings.

## Introduction

In 1881, Carl Wernicke described acute encephalopathy as characterized by mental confusion, ophthalmoplegia, nystagmus and gait ataxia. A few years later, a Russian psychiatrist named Sergei Korsakoff extended this syndrome by including memory loss and confabulation as the subsequent neuropsychiatric manifestations of the illness now known as Wernicke-Korsakoff syndrome (WKS) [1]. Wernicke encephalopathy (WE) is an acute neurological emergency resulting from a deficiency of thiamine (vitamin B1), the most common cause of which is alcohol abuse; however, prolonged starvation, hyperemesis gravidarum and gastrointestinal surgery can also lead to WE [2]. The prevalence of WE in the general population is about 0.4% to 2.8% but can be as high as 12.5% in alcohol abusers and up to 59% in alcohol-related deaths [2-3]. A pathological examination of acute lesions demonstrates vascular congestion with or without petechial haemorrhages, while chronic lesions can be demyelinating and gliotic with atrophy of the mammillary bodies being highly specific to WKS [4]. WE remains a clinical diagnosis which can be made even with normal blood thiamine levels and with magnetic resonance imaging (MRI) having a sensitivity and specificity of 53% and 93%, respectively [5]. The diagnosis of non-alcoholic WE seems trickier due to its atypical course. We herein described a non-alcoholic woman with an atypical clinical presentation but typical MRI findings [6-7].

## Case presentation

**Figure 1:**
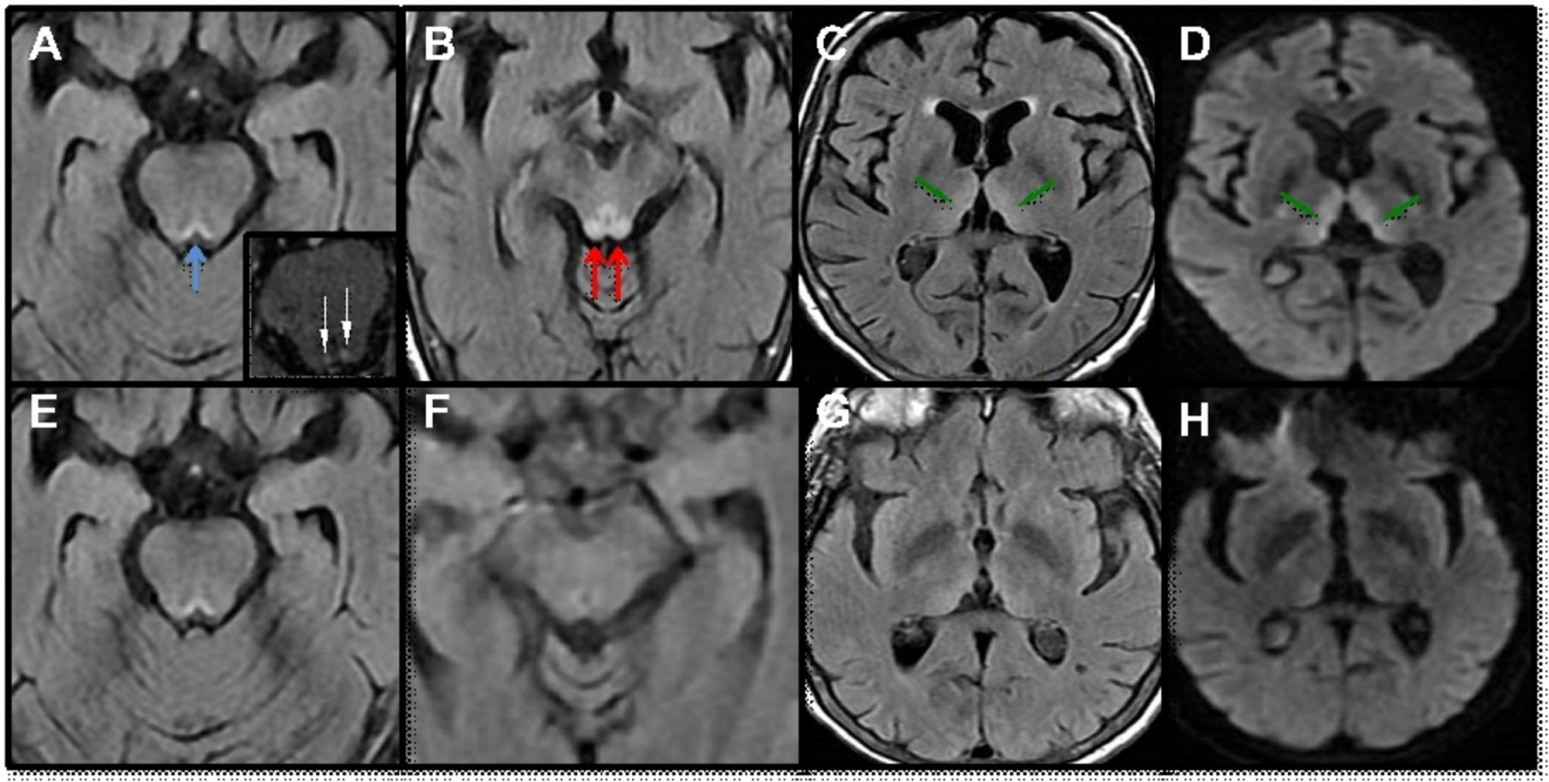
Changes on MRI with Wernicke's encephalopathy MRI brain with contrast showing T2 FLAIR signal hyperintensity involving the inferior tectal plate (A, blue arrow) with enhancement (A, inset) as well as the periaqueductal gray matter within the dorsal midbrain (B, red arrow). There is also involvement of the the medial thalami bilaterally (green arrow) on T2 FLAIR (C) as well as on DWI (D). Patient was treated with high dose intravenous thiamine with repeat MRI brain, one week later showing a significant improvement (E, F, G and H) MRI: magnetic resonance imaging, DWI: diffusion-weighted imaging

A 63-year-old woman, with a history of Boerhaave's oesophageal rupture status post-oesophagectomy with extra-thoracic transverse colon interposition 40 years ago, presented with two weeks of malaise and was found to have a non-mechanical primary bowel dysmotility. She was dehydrated and resuscitated in the emergency room with intravenous (IV) normal saline which was later switched to IV 5% dextrose normal saline. Over the next week, she became progressively more lethargic and was thought to be delirious with “sundowning”. On neurologic examination, she was drowsy but arousable to verbal stimuli with bilateral vertical and horizontal ophthalmoplegia. An MRI of the brain showed symmetrical hyperintensity in the bilateral medial thalami and dorsal midbrain, including periaqueductal grey matter, as shown in Figure 1. She was started on high-dose IV thiamine (500 mg three times daily for two days followed by 250 mg daily for five days followed by oral treatment with 100 mg daily) along with other B-complex vitamins and magnesium. Repeat brain MRI one week later showed significantly reduced hyperintensities in the thalamus and the dorsal midbrain region. Unfortunately, her hospital course was complicated by *Pseudomonas* and *Klebsiella pneumonia* requiring intubation, along with sepsis and progressive multiple organ failure. She passed away after the goal of her care was changed to comfort measures only.

## Discussion

Non-alcoholic WE was first described by Carl Wernicke himself in 1881 in a woman with pyloric stenosis and persistent vomiting from sulphuric acid ingestion who, on autopsy, was found to have bilateral punctate haemorrhages along the wall of the third ventricle [8]. He described a clinical triad of acute encephalopathy, ophthalmoplegia/nystagmus and gait ataxia. In 1997, Caine et al. suggested that a diagnosis of WE can be made if two of the four criteria are satisfied: (1) eye signs, (2) cerebellar signs, (3) memory impairment or confusion and (4) evidence of malnutrition on physical examination or from laboratory data [9]. Confusion is the most common presenting symptom occurring in about 82% of cases, followed by oculomotor abnormalities in 29%, gait ataxia in 23% and polyneuropathy in 11% [10]. Atypical clinical presentations include stupor, hypotension, vestibular dysfunction without hearing loss and beriberi heart disease that has been rarely reported with WE. While alcohol abuse remains the most common precipitating factor for WE, non-alcoholic aetiologies include starvation, hyperemesis gravidarum, IV infusion of glucose before thiamine, prolonged chemotherapy (5-fluorouracil (FU) infusion, doxyfluridine and ifosfamide), dialysis (thiamine is water soluble), anorexia nervosa, refeeding syndrome and gastrointestinal surgery. Thiamine is a water-soluble vitamin absorbed in the duodenum and stored primarily in the liver with body stores typically lasting up to 18 days [11]. The absorption of thiamine occurs in the duodenum by a carrier-mediated process (inhibited by alcohol via interference of intestinal ATPase) that requires magnesium as a cofactor; hence, hypomagnesaemia can mimic thiamine deficiency [11]. Thiamine plays a significant role in the Krebs cycle and pentose phosphate pathways. Thus, a deficiency in thiamine typically affects the metabolically active regions, producing vascular congestion with or without petechial haemorrhages in the acute phase and demyelinating, gliotic lesions in the chronic phase [4]. The prevalence of WE in patients with bariatric surgery is reported to be ~8% [12]. Singh et al. performed a systematic analysis of WE cases post bariatric surgery and found that out of the 32 cases diagnosed at 2–18 weeks from the procedure, the majority presented with the classic triad of WE, while nearly 50% had a normal MRI [13]. WE remains a clinical diagnosis that can be made even with normal blood thiamine levels and with MRI having a sensitivity and specificity of 53% and 93%, respectively [5]. Only 16% of the patients eventually diagnosed with WE, however, presented with the typical clinical triad, while about 19 % of the patients had none of the triad symptoms. Up to 80% of cases get missed during a routine clinical examination, and autopsy-based studies indicate that almost 82% of the patients with WE present with delirium [10]. While a presumptive diagnosis can be made by measuring the erythrocyte transketolase activity, it is limited by its lack of specificity and cost. To overcome this problem in diagnosis, consistent efforts have been undertaken in using neuroimaging techniques to support the clinical presentation of WE; however, treatment should not be delayed [10]. MRI lesions classically involve the medial thalami, especially along the third ventricular wall (80% to 85%), periaqueductal areas (59% to 65%), mammillary bodies (38% to 45%), tectal plate (36% to 38%) and dorsal medulla with the involvement of hypoglossal nuclei (8%) [10]. Other less common sites are the corpus callosum, caudate nucleus, red nucleus, dentate nucleus, cerebellum, hippocampus and frontal and parietal cerebral cortices. These regions are more prone to being sensitive to thiamine deficiency. Gross haemorrhage is uncommon in acute WE, but it has been reported on CT scans [10]. Prompt administration of thiamine leads to an improvement in the ocular signs within hours to days, vestibular function and gait ataxia during the second week, and confusion subsides over days to weeks. Abnormal T2 signal disappears as early as two days after treatment with thiamine; in our specific case, we observed a significant radiographic resolution within a week of starting thiamine.

## Conclusions

With the advancements in the neuroimaging techniques, MRI findings are useful in the early diagnosis of WE, especially with non-alcoholic cases with atypical clinical presentations. One must maintain a high index of suspicion for non-alcoholic WE, as a prompt initiation of treatment can significantly reduce long-term morbidity and mortality.
